# Circulating Tumor Cells: Isolation, Preclinical Models, and Clinical Applications for Personalized Cancer Therapy

**DOI:** 10.3390/biom16030394

**Published:** 2026-03-05

**Authors:** Luisana Sisca, Mariam Grazia Polito, Michele Iuliani, Giuseppe Francesco Papalia, Giuseppe Tonini, Francesco Pantano

**Affiliations:** 1 Department of Medical Oncology, Fondazione Policlinico Universitario Campus Bio-Medico, 00128 Rome, Italym.iuliani@policlinicocampus.it (M.I.);; 2 Department of Translational Research, Institut Curie, 75005 Paris, France; 3 UOC Oncologia Territoriale-ASL Latina-CDS Aprilia, La Sapienza Università Di Roma Polo Pontino, 04100 Latina, Italy; 4 Oncological Orthopedics Department, IFO-IRCCS Regina Elena National Cancer Institute, Via Elio Chianesi, 53, 00144 Rome, Italy

**Keywords:** circulating tumor cells, liquid biopsy, preclinical models, personalized cancer therapy, tumor heterogeneity, functional drug testing

## Abstract

Circulating tumor cells (CTCs) represent a powerful, minimally invasive window into tumor biology and disease evolution. Technological progress over the past decade has markedly improved the ability to isolate, preserve, and interrogate viable CTCs, transforming them from simple prognostic markers to functional tools for precision oncology. Advances in microfluidic platforms, immunomagnetic enrichment, aptamer-based capture, and nanostructured interfaces have expanded the efficiency and fidelity of CTC recovery, enabling comprehensive molecular profiling and ex vivo analysis. These innovations have paved the way for the development of CTC-derived preclinical models, including xenografts, organoids, and chorioallantoic membrane assays, which recapitulate patient-specific tumor heterogeneity and support individualized drug-sensitivity testing. In this review, we summarize current technologies for CTC isolation, outline recent achievements in functional and pharmacological characterization, and discuss the translational impact of CTC-derived models. We further identify persistent challenges and emerging opportunities, highlighting how integration of multi-omics platforms, artificial intelligence, and standardized workflows may accelerate the clinical implementation of CTC-guided personalized therapy.

## 1. Introduction

Liquid biopsy refers to the analysis of tumor-derived material released into the bloodstream, including circulating tumor cells (CTCs), circulating tumor DNA (ctDNA), extracellular vesicles, and other soluble components. Unlike conventional tissue biopsy, which provides a static and spatially limited snapshot of tumor biology, liquid biopsy enables minimally invasive, repeatable, and longitudinal monitoring of tumor evolution [1]. By capturing dynamic molecular changes that occur under therapeutic pressure, liquid biopsy approaches offer the potential to better reflect intratumoral heterogeneity and emerging resistance mechanisms.

Among the various liquid biopsy components, circulating tumor cells occupy a unique position. As intact and viable tumor cells, CTCs provide not only genomic and transcriptomic information but also functional and phenotypic insights that cannot be obtained from acellular biomarkers alone [2].

Circulating tumor cells, rare cells that detach from primary or metastatic tumors and enter the bloodstream, are at the forefront of cancer research due to their potential as non-invasive biomarkers. Despite their low abundance, often only a few cells per milliliter of blood, CTCs carry a wealth of biological information, including genetic, epigenetic, and phenotypic traits of the tumor from which they originate. The ability to isolate, characterize, and study these cells opens the door to understanding metastasis, assessing prognosis, and evaluating therapeutic response in real time [3].

Over the years, the field has evolved from simple enumeration of CTCs to sophisticated functional analyses. Early studies focused on capturing EpCAM-positive cells, providing initial evidence that CTC counts correlate with patient outcomes in breast, colorectal, lung, and prostate cancers. However, the recognition that CTCs are highly heterogeneous, including cells undergoing epithelial-to-mesenchymal transition (EMT) or expressing stem-like traits, has prompted the development of more refined isolation methods capable of preserving cell viability and function. These methodological advances, coupled with the creation of CTC-derived preclinical models, allow not only correlative studies but also predictive testing of drug responses, moving the field toward true personalized oncology [4]. From a molecular perspective, circulating tumor cells represent a unique system in which genomic instability, transcriptional plasticity, epigenetic regulation, and adaptive signaling programs can be studied at single-cell resolution. Unlike tissue biopsies, CTCs enable the longitudinal interrogation of dynamic molecular states that evolve under therapeutic pressure.

In this review, we aim to provide a comprehensive overview of current CTC isolation technologies, discuss advances in functional and pharmacological characterization, examine the development of CTC-derived preclinical models, and critically analyze the clinical challenges and future perspectives that will shape the integration of CTCs into personalized cancer therapy.

Key practice-defining and concept-shaping studies in CTC research are summarized in Table 1.

This table highlights selected studies that have shaped the translational and clinical development of circulating tumor cells, spanning isolation strategies, functional characterization, preclinical modeling, and clinical application. Rather than providing an exhaustive overview, the table focuses on practice- and concept-defining works that illustrate the evolution of CTCs from prognostic biomarkers to dynamic tools for personalized cancer therapy.

### CTCs Within the Liquid Biopsy Landscape

While circulating tumor cells represent a central focus of this review, other components of liquid biopsy, including circulating tumor DNA (ctDNA) and extracellular vesicles (EVs), have gained significant clinical relevance. ctDNA provides highly sensitive detection of tumor-specific genomic alterations and is particularly valuable for mutation profiling and minimal residual disease monitoring. However, as a fragmented acellular biomarker, ctDNA does not allow functional analyses or direct phenotypic characterization [12].

Extracellular vesicles, including exosomes, carry nucleic acids, proteins, and lipids reflective of tumor biology and may contribute to intercellular communication within the tumor microenvironment. Nevertheless, challenges related to isolation specificity and standardization remain.

**Figure 1:**
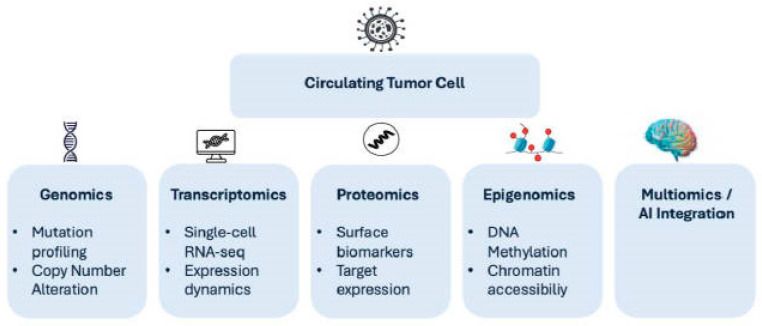
Multi-omics characterization of circulating tumor cells and integration into precision oncology.

In contrast, CTCs are intact, viable tumor cells that enable integrated genomic, transcriptomic, proteomic, and functional analyses at the single-cell level. Importantly, CTCs allow phenotypic characterization, assessment of protein expression (e.g., therapeutic targets), and the generation of ex vivo and in vivo models. A comparative overview of CTCs, ctDNA, and EVs as liquid biopsy components is summarized in Table 2. These unique features position CTCs as a complementary and potentially integrative component of liquid biopsy strategies in precision oncology [13]. The conceptual positioning of CTCs within the broader liquid biopsy framework is depicted in Figure 1.

Conceptual framework illustrating the integrative multi-omics analysis of circulating tumor cells (CTCs). Isolated CTCs undergo genomic (mutation profiling and copy number alterations), epigenomic (DNA methylation and chromatin accessibility), transcriptomic (single-cell RNA sequencing and dynamic expression programs), and proteomic (surface biomarker and target expression) characterization. Integration of these molecular layers through advanced computational and artificial intelligence–driven approaches enables identification of clinically actionable vulnerabilities. Functional validation using CTC-derived models further supports therapeutic decision-making, ultimately contributing to precision oncology strategies.

## 2. Technological Advances in CTC Isolation

The isolation of CTCs has been a central technical challenge, and the last decade has witnessed an explosion of innovative approaches. Broadly, methods can be categorized into those exploiting physical properties and those targeting biochemical characteristics of CTCs [14].

Physical property-based methods leverage differences in size, deformability, and density between CTCs and normal blood cells. Microfluidic platforms have become especially prominent. Spiral microchannels, microfilters, and deterministic lateral displacement devices allow the continuous, label-free separation of CTCs with minimal mechanical stress, thereby maintaining viability [15]. Dielectrophoresis (DEP) and ferrofluid-assisted separation offer alternative physical approaches, using electric or magnetic forces to manipulate cells based on dielectric or magnetic properties [16].

Biochemical approaches rely on the expression of specific surface markers. Immunomagnetic separation using antibodies against EpCAM or tumor-specific antigens remains widely used. However, the heterogeneity of CTCs, particularly those undergoing EMT, has driven the exploration of aptamer-based capture, where synthetic DNA or RNA sequences selectively bind target molecules with high specificity [17]. Nanostructured substrates, SERS (surface-enhanced Raman scattering), and photoelectrochemical (PEC) devices further enhance capture efficiency and allow simultaneous molecular characterization, often at the single-cell level [18].

Equally important is the release and reculture of captured CTCs. Maintaining cell viability is critical for downstream functional analyses, including drug sensitivity testing and preclinical modeling. Strategies such as thermoresponsive coatings, reversible binding chemistries, and gentle enzymatic release have been developed to maximize recovery of live CTCs without compromising their biological properties [19]. Importantly, the choice of CTC isolation technology directly influences the type and quality of downstream molecular information that can be obtained, ranging from genomic and transcriptomic profiling to proteomic and functional analyses at the single-cell level.

In parallel with experimental enrichment strategies, several CTC isolation technologies have reached commercial development. The CellSearch^®^ system remains the only FDA-cleared platform for CTC enumeration in selected tumor types and relies on EpCAM-based immunomagnetic capture. While clinically validated, its epithelial marker dependence may limit the detection of mesenchymal-like CTCs. Label-free approaches such as Parsortix^®^ and ClearCell^®^ exploit physical properties including cell size and deformability, enabling enrichment of viable CTCs suitable for downstream molecular and functional analyses [20]. Additional platforms, including VTX-1 (hydrodynamic vortices), RareCyte^®^ (integrated immunocapture and imaging), and IsoFlux^®^ (microfluidic immunomagnetic enrichment), further illustrate the technological diversity of the field. Despite these advances, variability in capture efficiency, phenotypic bias, and downstream compatibility underscores the need for cross-platform standardization and validation [21]. A comparative overview of the main experimental and commercial CTC isolation platforms is provided in Table 3.

Summary of selected commercially developed CTC enrichment technologies, highlighting their isolation principles, marker dependence, ability to recover viable cells, regulatory status, strengths, and limitations. Platforms differ in their reliance on epithelial markers, physical properties, or hybrid approaches, influencing capture efficiency, phenotypic bias, and suitability for downstream molecular and functional analyses. While CellSearch^®^ remains the only FDA-cleared system for CTC enumeration in selected tumor types, newer label-free and microfluidic platforms enable viable cell recovery, supporting multi-omics profiling and functional testing. However, variability across technologies underscores the ongoing need for cross-platform standardization and clinical validation.

Recent efforts have also been aimed at capturing true live single CTCs with minimal leukocyte contamination, a prerequisite for reliable downstream multi-omics profiling. Emerging ligand-based bead platforms have demonstrated high analytical sensitivity and specificity in large pan-cancer cohorts, enabling live CTC isolation compatible with genomic and proteomic analyses. A late-breaking AACR 2025 [22] report described a single-CTC capture system with high capture efficiency and minimal false positives, highlighting the potential of contamination-free CTC isolation for comprehensive molecular profiling. While further peer-reviewed validation is required, these advances reflect the rapid evolution of technologies supporting viable CTC recovery and multi-omics applications 

## 3. Functional and Pharmacological Characterization of CTCs

The true power of CTC research lies in functional analysis. Beyond enumeration, CTCs are studied to reveal their phenotypic diversity and to probe drug sensitivity. Multi-parametric approaches allow characterization of EMT markers, apoptosis, proliferation, and key oncogenic signaling pathways. Single-cell analyses have uncovered rare subpopulations with stem-like or therapy-resistant features, which may drive metastasis and relapse [23].

Pharmacological testing on CTCs or CTC-derived cultures has demonstrated predictive potential. For instance, studies have shown differential sensitivity of CTCs to platinum-based agents, PARP inhibitors, BCL-2 inhibitors, and EGFR-targeted therapies, often correlating with clinical response. These findings highlight how functional profiling of CTCs can provide actionable information, potentially guiding individualized therapy [24].

## 4. Preclinical Models Derived from CTCs

One of the most exciting developments in the field is the generation of preclinical models from CTCs, which bridge the gap between patient biology and experimental testing.

CTC-derived xenografts (CDXs) involve implanting patient CTCs into immunodeficient mice. These models faithfully recapitulate tumor heterogeneity, metastatic potential, and therapy response, providing a personalized platform for drug evaluation. Similarly, patient-derived xenografts (PDXs), while often originating from tumor tissue rather than CTCs, complement CDXs by offering comparative data on tumor biology and drug sensitivity [9].

Organoids, three-dimensional cultures derived from CTCs, preserve tissue architecture and enable high-throughput pharmacological testing. These systems can maintain the molecular and functional heterogeneity of tumors, offering insights into resistance mechanisms. Chorioallantoic membrane (CAM) assays provide a rapid, cost-effective model to assess tumor growth, angiogenesis, and drug response, allowing preliminary screening before more resource-intensive studies [11].

**Figure 2:**
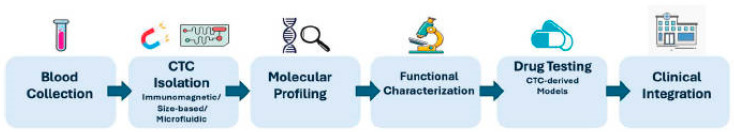
Translational workflow of circulating tumor cell (CTC) analysis in precision oncology.

Collectively, these models provide a continuum from CTC isolation to functional and preclinical validation, facilitating a deeper understanding of tumor biology and therapy response. An overview of the CTC translational workflow from isolation to clinical application is illustrated in Figure 2. These CTC-derived models provide an opportunity not only for pharmacological testing but also for dissecting patient-specific molecular dependencies, signaling vulnerabilities, and adaptive resistance mechanisms.

Schematic representation of the CTC-based translational pipeline. Peripheral blood collection is followed by CTC isolation using immunomagnetic, size-based, or microfluidic approaches. Isolated viable CTCs undergo comprehensive molecular profiling, including genomic, transcriptomic, and proteomic analyses, with integration of multi-omics data and surface biomarker assessment. Functional characterization, such as drug sensitivity testing and development of CTC-derived models, enables evaluation of therapeutic vulnerabilities. Ultimately, integration of longitudinal CTC data into clinical practice supports prognosis assessment, real-time therapy monitoring, and identification of actionable targets, advancing personalized cancer therapy.

## 5. Clinical Applications of CTCs

The incorporation of circulating tumor cells into clinical oncology represents one of the most intriguing attempts to bridge the gap between the dynamic biology of cancer and the static nature of our current diagnostic tools. In everyday practice, oncologists must make therapeutic decisions based on information that is inherently fixed in time: a biopsy obtained months earlier, imaging studies that reveal only the macroscopic surface of the disease, or a molecular profile that captures just one snapshot of an evolving malignancy. CTCs, in contrast, offer a direct view of the “tumor in motion,” a real-time portrait of what the disease has become at the very moment we are treating it [25].

From a prognostic standpoint, their utility is well established. CTC enumeration consistently identifies patients with more aggressive disease, often outperforming conventional biomarkers in predicting progression [5,6]. Yet focusing solely on enumeration greatly underestimates their potential. The true transformative capacity of CTCs lies not in counting them, but in watching them change. The emergence of mesenchymal traits, the appearance of stem-like subclones, or the activation of survival pathways can all precede radiographic or clinical evidence of resistance. These biological signals offer a temporal advantage that our traditional tools cannot match.

Despite this, the distance between what CTCs can reveal and what clinicians can act upon remains substantial. Detecting an EMT shift or the rise of a resistant subclone may alert the oncologist but rarely leads to immediate therapeutic modification. This is not due to a limitation of the cells themselves, but rather of our clinical frameworks: validated decision algorithms are lacking, and prospective trials demonstrating that CTC-guided interventions improve survival are still missing [7]. In essence, we are capable of reading the story CTCs tell, but we have not yet learned how to rewrite the ending.

The therapeutic implications are equally compelling. Molecular characterization of CTCs, identifying driver mutations, amplifications, or resistance mechanisms, raises important clinical questions. If a primary tumor was HER2-negative but CTCs now exhibit HER2 amplification, which biology should guide therapy: the tumor of the past or the tumor that is emerging now? CTCs thus challenge the notion of a “definitive” molecular profile and highlight the need for dynamic biomarkers that evolve with the disease [10].

Even more ambitious is the development of CTC-derived functional models. Culturing organoids or generating xenografts from bloodborne tumor cells represents one of the most visionary frontiers of personalized oncology [8]. In principle, clinicians could test therapeutic agents ex vivo on a patient’s own circulating cells, obtaining a preview of drug sensitivity before exposing the patient to toxicity. However, this paradigm faces practical barriers: insufficient numbers of viable CTCs, variable success in establishing models, the time required for expansion, and the challenge of integrating results into time-sensitive treatment decisions. The concept is revolutionary, yet its clinical impact is still emerging.

Thus, CTCs now occupy a compelling middle ground: too informative to ignore, yet not fully ready for routine implementation. They offer an extraordinarily precise window into real-time tumor evolution but also remind us that biological insight alone is not enough. To truly alter clinical practice, we must learn how to translate their dynamic information into actionable therapeutic strategies.

## 6. CTC Heterogeneity and Biological Subpopulations

Circulating tumor cells are not a uniform population but rather a highly heterogeneous ensemble of cells that reflect the evolutionary complexity of the tumor from which they originate [26]. This heterogeneity encompasses phenotypic, molecular, and functional dimensions and represents one of the defining features that both enriches the biological relevance of CTCs and complicates their clinical interpretation. Understanding the diversity of CTC subpopulations is therefore essential to fully appreciate their role in metastasis, therapy resistance, and disease progression [27].

One of the earliest recognized axes of CTC heterogeneity lies along the epithelial–mesenchymal spectrum. While initial detection platforms were optimized to capture epithelial CTCs expressing markers such as EpCAM and cytokeratins, it has become increasingly evident that a substantial fraction of CTCs exhibits partial or complete mesenchymal traits [28]. These cells often display reduced epithelial marker expression, enhanced migratory capacity, and resistance to apoptosis, features associated with epithelial-to-mesenchymal transition (EMT). Importantly, EMT should not be viewed as a binary process but rather as a continuum, with many CTCs occupying hybrid epithelial/mesenchymal states. These intermediate phenotypes may confer maximal plasticity, enabling CTCs to survive in circulation while retaining the capacity to colonize distant organs [29]. This phenotypic plasticity is increasingly recognized as being supported by epigenetic reprogramming and dynamic transcriptional state transitions rather than fixed genetic alterations alone.

Beyond EMT, another biologically relevant subpopulation consists of stem-like CTCs. These cells express markers associated with self-renewal, pluripotency, and tumor-initiating capacity, and are thought to play a central role in metastatic seeding and relapse [30]. Single-cell analyses have revealed that stem-like traits frequently coexist with mesenchymal features, suggesting that metastatic competence may arise from the convergence of multiple phenotypic programs [31]. The presence of such rare but highly aggressive subclones within the circulating compartment underscores why simple CTC enumeration may fail to capture clinically meaningful information.

CTC heterogeneity is further amplified by the existence of CTC clusters, multicellular aggregates composed of tumor cells alone or in association with stromal or immune cells. Although numerically less abundant than single CTCs, clusters exhibit disproportionately high metastatic potential. Their collective migration confers protection from shear stress and immune surveillance, while intercellular interactions promote survival signaling and resistance to therapy. Molecular profiling of clusters has demonstrated distinct transcriptional programs compared with single CTCs, reinforcing the notion that they represent a biologically unique entity rather than a mere aggregation artifact.

Functional heterogeneity among CTCs also extends to their interaction with the tumor microenvironment and the immune system. Some CTCs display immune-evasive properties, such as altered antigen presentation or association with platelets and neutrophils, which may facilitate survival in the bloodstream. Others appear biologically inert, lacking the capacity to proliferate or seed metastases. This functional stratification highlights a critical challenge in the field: not all detected CTCs are clinically equivalent and collapsing them into a single category risks obscuring the contributions of biologically meaningful subpopulations.

From a translational perspective, CTC heterogeneity represents both an opportunity and a limitation. On one hand, it provides a dynamic snapshot of tumor evolution that cannot be captured by a single tissue biopsy. On the other hand, it complicates efforts to define standardized biomarkers or therapeutic thresholds. The development of functional assays and CTC-derived models offers a potential solution by allowing biological behavior, rather than marker expression alone, to guide interpretation. In this context, heterogeneity should not be regarded as a technical obstacle to overcome, but as a fundamental biological feature that must be integrated into the design of CTC-based clinical applications.

Beyond intrinsic molecular programs, accumulating evidence suggests that circulating tumor cell (CTC) heterogeneity and cluster formation are not purely stochastic phenomena, but are shaped by microenvironmental pressures. The primary tumor microenvironment, including hypoxia, stromal activation, and immune cell infiltration, may pre-condition subclones with enhanced plasticity, immune-evasive properties, and metastatic competence prior to intravasation. Once in circulation, CTCs interact dynamically with platelets, neutrophils, and endothelial cells, forming a transient “circulatory niche” that can promote survival and facilitate extravasation. Similarly, organ-specific metastatic niches may selectively support particular CTC phenotypes, contributing to organotropism and functional diversity. These observations suggest that CTC biology is influenced not only by intrinsic genomic alterations but also by ecological contexts, raising the possibility that modulation of the CTC niche could represent a complementary therapeutic strategy in precision oncology [32,33].

## 7. Methodological, Clinical, and Regulatory Challenges

Despite the rapid expansion of circulating tumor cell research and the increasing sophistication of isolation and characterization technologies, several critical challenges continue to limit the routine clinical implementation of CTC-based approaches. These challenges span methodological, clinical, and regulatory domains and must be carefully considered when interpreting current evidence and designing future studies.

From a methodological perspective, heterogeneity among isolation platforms remains one of the most significant barriers. CTC enrichment strategies differ substantially in their underlying principles, including size-based separation, immunoaffinity capture, and hybrid approaches, each introducing specific biases in the recovered cell populations. As a consequence, results obtained using different platforms are often not directly comparable. In addition, pre-analytical variables such as blood collection tubes, time to processing, storage conditions, and sample handling can markedly influence CTC yield, viability, and downstream analyses. The lack of standardized protocols across laboratories hampers reproducibility and complicates the translation of promising findings into multicenter clinical studies.

Sensitivity and scalability represent additional limitations. While advanced technologies can achieve high capture efficiency and preserve cell viability, many platforms remain technically demanding and resource-intensive. This restricts their availability to specialized centers and limits integration into routine oncology workflows. Moreover, the intrinsic rarity of CTCs, particularly in early-stage disease or in tumor types with low shedding rates, constrains the feasibility of extensive molecular or functional analyses. As a result, many functional assays and CTC-derived models are currently applicable only to selected patient subsets, often those with advanced or highly aggressive disease [34].

Clinical integration poses further challenges. Although the prognostic value of CTC enumeration is well established in several tumor types, the clinical utility of CTC-guided treatment decisions remains less clearly defined. Prospective interventional trials demonstrating improved patient outcomes based on CTC-driven therapeutic modifications are still limited. In routine practice, clinicians are often confronted with biologically informative CTC data without clear, validated algorithms translating these findings into actionable decisions. This gap between biological insight and clinical action represents one of the most pressing hurdles in the field.

Time constraints also play a critical role. Functional characterization of CTCs, including ex vivo drug testing or the generation of preclinical models, requires time for cell expansion and assay completion. In many clinical scenarios, particularly in rapidly progressing metastatic disease, treatment decisions must be made within narrow time windows that may not accommodate prolonged experimental workflows. Bridging this temporal mismatch will be essential for the successful clinical adoption of CTC-based functional approaches.

Regulatory considerations further shape the translational landscape. Most CTC isolation and analysis platforms are currently designated for research use only, and only a limited number have achieved regulatory approval for specific clinical applications. Demonstrating analytical validity, clinical validity, and clinical utility is particularly challenging for complex assays involving live-cell manipulation, multi-step processing, or functional testing. In addition, regulatory pathways for personalized ex vivo drug testing remain poorly defined, raising ethical and logistical questions regarding clinical implementation and reimbursement [35].

Finally, there is a broader conceptual challenge related to aligning technological innovation with clinically meaningful endpoints. The field has produced a wide array of highly sophisticated platforms, yet technical advancement alone does not guarantee clinical impact. Future progress will depend on integrated strategies that combine robust biological rationale, standardized methodologies, and carefully designed clinical trials focused on patient-centered outcomes. Addressing these challenges will be essential to move CTC-based approaches from exploratory research tools toward reliable components of precision oncology [36].

## 8. Trends, Gaps, and Future Directions

The field of circulating tumor cells is evolving with a dual momentum: technological innovation on one side, and clinical caution on the other. Recent years have brought an impressive expansion of platforms capable of isolating rare cells with a precision unimaginable a decade ago. Yet not all technological advances meaningfully shift the clinical landscape. Many platforms compete on engineering sophistication rather than addressing the fundamental question that defines clinical utility: will this improve patient outcomes?

The literature is extensive but often fragmented. Most studies focus on selected tumor types and endpoints that do not necessarily align with clinical decision-making. CTCs are frequently treated as a homogeneous population, even though their diversity reflects the evolutionary complexity of cancer far more accurately than any tissue biopsy. This oversimplification risks obscuring the biological narratives encoded within circulating cells. There is growing recognition that not all CTCs are equal; some may drive metastasis, others may be biologically inert, and collapsing them into a single metric may diminish interpretive power [37].

In parallel with technological advances, the establishment of standardized workflows and shared methodological frameworks remains a critical priority for circulating tumor cell research. Variability in isolation platforms, analytical pipelines, and reporting criteria continues to limit cross-study comparability and weaken clinical translation. Pre-analytical handling, capture efficiency, and downstream characterization significantly influence CTC readouts, often hindering inter-institutional validation. The development of consensus guidelines for analytical validation and data interpretation will therefore be essential to ensure reproducibility and regulatory credibility. Importantly, standardization should be regarded not as a constraint, but as a foundation for meaningful technological comparison and clinical implementation.

Large collaborative consortia and multicenter initiatives are likely to play a central role in this process. Coordinated efforts that integrate longitudinal CTC analyses with clinical outcomes across different tumor types and treatment settings could generate the high-quality datasets required to move the field beyond descriptive observations. Such initiatives would facilitate the identification of biologically and clinically relevant CTC subpopulations, clarify their temporal dynamics under therapeutic pressure, and support the development of robust predictive models. Moreover, shared infrastructures and harmonized data collection strategies would enhance statistical power and accelerate the translation of CTC-based findings into clinically actionable knowledge [38].

Another critical challenge for future implementation lies in aligning CTC-based technologies with the realities of clinical decision-making. While the biological promise of CTC analyses is widely recognized, their integration into routine oncology practice must account for clinical timelines, treatment urgency, and healthcare system constraints. Functional assays and CTC-derived preclinical models are particularly appealing, yet their current time requirements and variable success rates limit immediate applicability in rapidly evolving disease settings. Future efforts should therefore focus on streamlining analytical workflows, reducing turnaround times, and identifying clinical contexts in which CTC-guided strategies are most likely to provide tangible benefit. Rather than aiming for universal applicability, CTC-based approaches may initially find their greatest value in selected scenarios, such as treatment-resistant metastatic disease or cases characterized by molecular discordance between primary tumors and metastatic lesions [39].

Finally, the transition from exploratory research to routine clinical application will depend on the generation of high-level evidence demonstrating that CTC-informed interventions improve patient outcomes. While observational and correlative studies have established the prognostic relevance of CTCs, interventional trials remain relatively limited. Addressing this evidence gap will require carefully designed clinical studies that explicitly test whether modifying therapeutic strategies based on CTC-derived information leads to meaningful clinical benefit. The success of this translational step will ultimately determine whether circulating tumor cells evolve from powerful research tools into reliable components of personalized cancer care [40]. Compounding this is the challenge of generating robust, integrated, longitudinal datasets. Truly unlocking the potential of CTCs would require systematic collection of genomic, transcriptomic, proteomic, epigenetic, and clinical data over time, an endeavor that few centers are currently equipped to execute at scale. Without such datasets, the promise of artificial intelligence remains largely theoretical: algorithms do not lack sophistication; they lack the depth and quality of data necessary to learn. In this context, the primary value of artificial intelligence lies not merely in outcome prediction but in the integration of multi-omics CTC data to infer regulatory networks, signaling hierarchies, and molecular trajectories underlying tumor evolution.

Another underexplored dimension concerns organotropism. Metastasis does not unfold randomly; it follows biological rules shaped by the intrinsic properties of specific CTC subpopulations. Cells that seed liver metastases differ substantially from those that colonize bone or brain. Ignoring this diversity means overlooking the very mechanisms that ultimately determine prognosis.

Most importantly, the field faces a critical validation gap. While descriptive and observational studies abound, interventional trials remain rare. The central question, whether modifying treatment based on CTC-derived information improves patient outcomes, remains unanswered [41]. Until this evidence is produced, clinical adoption will remain limited to research centers and early adopters rather than becoming a standard component of oncology practice.

Despite these challenges, the trajectory of the field is undeniably forward. As technology becomes more integrated, biological interpretation more refined, and clinical frameworks more adaptive, CTCs have the potential to reshape how we understand and manage metastatic disease. They may ultimately serve as one of the most powerful tools available for anticipating tumor evolution and guiding personalized therapy. Whether this potential becomes reality depends on our ability to harness its complexity and translate it into meaningful clinical action. In addition, future progress in the field will depend on the ability to integrate circulating tumor cell analyses with other components of liquid biopsy, including circulating tumor DNA, extracellular vesicles, and immune profiling. Rather than competing technologies, these approaches should be viewed as complementary tools that capture distinct dimensions of tumor biology [42]. Multimodal strategies combining cellular and acellular biomarkers may provide a more comprehensive and dynamic representation of tumor evolution, treatment response, and resistance mechanisms. Such integrative frameworks are likely to enhance predictive accuracy and support more informed therapeutic decision-making, further strengthening the role of CTCs within personalized oncology. In parallel, increasing attention is being devoted to the real-time assessment of CTC surface biomarkers, which may enable dynamic companion diagnostics and more adaptive therapeutic decision-making [43].

Beyond technological and methodological considerations, the future clinical impact of circulating tumor cells will also depend on how effectively their biological complexity can be translated into clinically meaningful stratification tools. While CTC analyses provide unparalleled insight into tumor evolution, the challenge lies in distinguishing descriptive richness from actionable relevance. Not all molecular alterations or phenotypic shifts observed in circulating cells will necessarily warrant therapeutic intervention, and defining thresholds for clinical action remains an open question. This underscores the need for interpretive frameworks that prioritize biological signals with demonstrated prognostic or predictive value.

An additional dimension that warrants further exploration is the temporal dynamics of CTC populations. Longitudinal sampling enables the monitoring of tumor evolution under therapeutic pressure, offering a unique opportunity to capture early adaptive responses that precede overt clinical progression. However, translating these dynamic changes into treatment modifications requires careful consideration of timing, magnitude, and clinical context. Overinterpretation of transient or subclonal signals may lead to premature or inappropriate therapeutic adjustments, highlighting the importance of integrating CTC-derived data with radiological, clinical, and other molecular biomarkers.

From a systems perspective, the integration of CTC analyses into multidisciplinary clinical workflows represents both a logistical and cultural challenge. Effective implementation will require close collaboration between oncologists, pathologists, translational researchers, and bioinformaticians, as well as the development of reporting formats that are interpretable and clinically intuitive. Standardized reporting of CTC characteristics, coupled with decision-support tools, may facilitate the incorporation of complex biological data into routine practice without overwhelming clinicians [44].

Ultimately, the long-term success of CTC-based approaches will depend on their ability to demonstrate added value beyond existing diagnostic and monitoring tools. As the field matures, emphasis should shift toward identifying specific clinical scenarios in which CTC analyses provide unique and actionable insights, rather than pursuing broad applicability across all disease settings. By aligning biological sophistication with clinical pragmatism, circulating tumor cells may evolve from powerful research instruments into targeted tools that meaningfully inform personalized cancer care [45].

In this context, it is also important to consider the educational and infrastructural requirements associated with the broader adoption of CTC-based technologies. The successful integration of complex liquid biopsy tools into clinical practice will depend not only on technological maturity but also on adequate training of clinical teams and the availability of dedicated laboratory and bioinformatics support. As precision oncology continues to evolve, fostering cross-disciplinary expertise and shared interpretive frameworks will be essential to ensure that increasingly sophisticated biological data can be translated into clear, timely, and clinically relevant information for patient management.

Beyond enumeration and genomic profiling, the characterization of surface biomarkers on circulating tumor cells has gained increasing clinical relevance. The dynamic assessment of therapeutic targets, including PD-L1, HER2, and AR-V7, directly on CTCs provides an opportunity to monitor treatment responsiveness and emerging resistance mechanisms in real time [46].

Unlike static tissue biopsies, CTC-based surface marker analysis enables longitudinal evaluation of phenotypic shifts under therapeutic pressure. For instance, changes in HER2 expression or androgen receptor splice variants detected on CTCs may inform treatment adaptation in breast and prostate cancer, respectively. Moreover, the integration of CTC surface biomarker profiling into companion diagnostic strategies represents a promising avenue for personalized therapy selection [47].

However, variability in detection platforms, antibody specificity, and marker heterogeneity across CTC subpopulations remain challenges that require further standardization before routine clinical implementation.

## 9. Multi-Omics Profiling of Circulating Tumor Cells: Advances and Challenges

Recent technological advances have enabled the application of multi-omics approaches to circulating tumor cells, allowing integrative characterization at the single-cell level. Single-cell genomic analyses have improved the detection of actionable mutations, copy number alterations, and clonal heterogeneity. In parallel, single-cell transcriptomic profiling has provided insights into dynamic gene expression programs associated with epithelial–mesenchymal transition, therapy resistance, and metastatic potential [48].

Recent single-CTC genomic studies further illustrate the potential of multi-omics interrogation to uncover clinically relevant heterogeneity. In a metastatic lung cancer cohort, targeted profiling of individual viable CTCs revealed multiple pathogenic variants, including alterations not detected in matched plasma ctDNA, highlighting inter- and intra-patient genomic diversity and potential early resistance signatures [49]. Notably, comparative analyses suggested distinct patterns of genome stability indicators between single CTCs and bulk ctDNA, underscoring the complementary biological information provided by cellular versus acellular liquid biopsy components. However, these studies were conducted in limited cohorts and remain technically demanding, reinforcing the need for standardized workflows and large-scale validation. Comparative features of circulating tumor cells (CTCs), circulating tumor DNA (ctDNA), and extracellular vesicles (EVs) as liquid biopsy components.

Proteomic characterization of CTCs, including assessment of surface biomarkers and therapeutic targets, further enhances the translational relevance of these analyses. Importantly, the integration of genomic, transcriptomic, and proteomic layers offers a more comprehensive understanding of tumor evolution under treatment pressure.

Despite these advances, several technical and biological challenges remain. The extreme rarity of CTCs in peripheral blood limits input material and increases susceptibility to technical noise and amplification bias. Single-cell sequencing approaches are prone to dropout events and uneven coverage, potentially affecting data interpretation [50]. Moreover, the lack of standardized isolation protocols and cross-platform validation hampers reproducibility across studies.

From a clinical perspective, cost, scalability, and turnaround time remain critical barriers to routine implementation. The integration of multi-omics datasets also requires advanced computational frameworks and robust bioinformatic pipelines to extract clinically actionable information.

Future efforts should focus on harmonizing isolation methods, improving single-cell sensitivity, and developing standardized analytical pipelines. The convergence of multi-omics technologies with artificial intelligence-driven data integration may ultimately enable CTC-based precision oncology strategies that are both biologically informative and clinically feasible [51].

## 10. Conclusions

Looking forward, the integration of multi-omics technologies with CTC analysis is expected to play a pivotal role in advancing precision oncology. The ability to combine genomic, transcriptomic, proteomic, and functional data from single circulating tumor cells may provide a more comprehensive understanding of tumor evolution and therapeutic vulnerabilities. However, the clinical translation of these approaches will depend not only on technological refinement but also on harmonized methodological frameworks.

A major unmet need in the field remains the establishment of industry-wide consensus on CTC isolation, enumeration, and downstream analytical pipelines. Variability across platforms and a lack of standardized validation criteria currently limit cross-study comparability and regulatory implementation. Collaborative efforts between academia, industry, and regulatory bodies will be essential to define reproducible standards and enable broader clinical adoption of CTC-based diagnostics.

Ultimately, the convergence of technological innovation, computational integration, and standardized methodologies will determine whether CTC multi-omics can transition from promising research tools to routine components of personalized cancer therapy.

## Figures and Tables

**Table 1:** Landmark studies in circulating tumor cell research.

Study (Year)	Tumor Type	CTC Isolation Strategy	Downstream Application	Key Finding/Clinical Relevance
Bidard et al. (2013–2021) [5,6,7]	Breast cancer	EpCAM-based enrichment	Enumeration/therapy guidance	CTC count predicts prognosis and guides treatment selection
Baccelli et al. (2013) [8]	Breast cancer	Immunomagnetic isolation	CDXs	Identification of metastasis-initiating CTC subpopulation
Kahounová et al. (2023) [9]	Multiple	Microfluidics/label-free	CDX, organoids	Feasibility of CTC-derived preclinical models
Lin et al. (2021) [4]	Pan-cancer	Multiple platforms	Molecular profiling	Demonstrated biological heterogeneity of CTCs
Gerratana et al. (2025) [10]	Breast cancer	CellSearch + molecular assays	Treatment monitoring	Dynamic molecular evolution captured by CTCs
Peng et al. (2025) [11]	Solid tumors	Viability-preserving capture	Organoids/drug testing	Functional drug screening from liquid biopsy
Bidard et al. (STIC-CTC trial) [7]	Breast cancer	CellSearch	Interventional trial	CTC-driven therapy non-inferior to clinician-driven choice

**Table 2:** Comparative features of circulating tumor cells (CTCs), circulating tumor DNA (ctDNA), and extracellular vesicles (EVs) as liquid biopsy components. Comparison of the main biological and clinical characteristics of circulating tumor cells (CTCs), circulating tumor DNA (ctDNA), and extracellular vesicles (EVs). While ctDNA provides highly sensitive genomic information and is widely applied for mutation profiling and minimal residual disease monitoring, it lacks phenotypic and functional insight. EVs enable multi-layer molecular analysis but remain limited by isolation specificity and standardization challenges. In contrast, CTCs, as intact viable tumor cells, uniquely allow integrated genomic, transcriptomic, proteomic, and functional characterization at single-cell resolution, supporting ex vivo modeling and precision oncology strategies. However, their rarity and technical complexity remain important limitations.

Feature	CTCs	ctDNA	Extracellular Vesicles (EVs)
Biological nature	Intact viable tumor cells	Tumor-derived DNA fragments	Membrane-bound vesicles containing nucleic acids and proteins
Genomic profiling	Yes	Yes	Yes (indirect)
Transcriptomic profiling	Yes	No	Yes
Proteomic profiling	Yes	No	Yes
Functional assays	Yes (ex vivo culture, CDX, organoids)	No	Limited
Single-cell resolution	Yes	No	Limited
Longitudinal monitoring	Yes	Yes	Yes
Clinical applications	Prognostic, predictive, functional modeling	Mutation profiling, minimal residual disease (MRD), treatment monitoring	Emerging diagnostic and monitoring tool
Main limitations	Rarity; technical complexity; heterogeneity	Fragmentation; lack of phenotypic and functional data	Isolation specificity; standardization challenges

**Table 3:** Comparative overview of commercially available circulating tumor cell (CTC) isolation platforms.

Platform	Isolation Principle	Marker Dependence	Recovery of Viable Cells	Regulatory Status	Main Strengths	Main Limitations
CellSearch^®^	EpCAM-based immunomagnetic enrichment	Yes (EpCAM-dependent)	Limited (fixed cells for enumeration)	FDA-cleared (breast, colorectal, prostate cancer)	Clinically validated; standardized enumeration	Misses EMT/mesenchymal CTCs; limited functional use
Parsortix^®^	Size and deformability-based microfluidic capture	No	Yes	CE-marked	Label-free enrichment; viable cells suitable for downstream assays	Size overlap with leukocytes; variable recovery
ClearCell^®^ FX	Inertial microfluidics (Dean flow fractionation)	No	Yes	Research use	Continuous flow; label-free; viable cell recovery	May lose smaller CTC subpopulations
VTX-1	Hydrodynamic vortex trapping	No	Yes	Research use	Gentle processing; preserves cell integrity	Limited large-scale validation
RareCyte^®^	Multi-marker immunofluorescence + imaging	Partial (multi-marker)	Yes (depending on workflow)	Research use	High-resolution imaging; phenotypic profiling	Complex workflow; limited standardization
IsoFlux^®^	Microfluidic immunomagnetic enrichment	Yes (antibody-dependent)	Yes	Research use	High sensitivity; customizable antibodies	Marker bias; less standardized clinically

## Data Availability

Not applicable.
